# The impact of prolonged, maternal iodine exposure in early gestation on neonatal thyroid function

**DOI:** 10.3389/fendo.2023.1080330

**Published:** 2023-01-31

**Authors:** Divya M. Mathews, Jane M. Peart, Robert G. Sim, Susannah O’Sullivan, José G. B. Derraik, Natasha L. Heather, Dianne Webster, Neil P. Johnson, Paul L. Hofman

**Affiliations:** ^1^ Liggins Institute, University of Auckland, Auckland, New Zealand; ^2^ Starship Children’s Hospital, Te Whatu Ora – Health New Zealand, Te Toka Tumai Auckland, Auckland, New Zealand; ^3^ Auckland Radiology Group, Auckland, New Zealand; ^4^ Endocrinology, Greenlane Clinical Centre, Te Whatu Ora – Health New Zealand, Te Toka Tumai Auckland, Auckland, New Zealand; ^5^ Department of Paediatrics: Child & Youth Health, Faculty of Medicine and Health Sciences, University of Auckland, Auckland, New Zealand; ^6^ Department of Women’s and Children’s Health, Uppsala University, Uppsala, Sweden; ^7^ Environmental-Occupational Health Sciences and Non-Communicable Diseases Research Group, Research Institute for Health Sciences, Chiang Mai University, Chiang Mai, Thailand; ^8^ Newborn Metabolic Screening Programme, Lab Plus, Te Whatu Ora – Health New Zealand, Te Toka Tumai Auckland, Auckland, New Zealand; ^9^ Robinson Research Institute, University of Adelaide, Adelaide, SA, Australia; ^10^ Department of Obstetrics and Gynecology, Faculty of Medical and Health Sciences, University of Auckland, Auckland, New Zealand; ^11^ Repromed Auckland, Auckland, New Zealand

**Keywords:** contrast, hypothalamic-pituitary axis, hypothyroidism, hysterosalpingography, iodine, newborn, oil-soluble, thyroid

## Abstract

**Context:**

Hysterosalpingography (HSG) using oil-soluble contrast medium (OSCM) improves pregnancy rates but results in severe and persistent iodine excess, potentially impacting the fetus and neonate.

**Objective:**

To determine the incidence of thyroid dysfunction in newborns conceived within six months of OSCM HSG.

**Design:**

Offspring study of a prospective cohort of women who underwent OSCM HSG.

**Setting:**

Auckland region, New Zealand (2020-2022)

**Participants:**

Offspring from the SELFI (Safety and Efficacy of Lipiodol in Fertility Investigations) study cohort (n=57).

**Measurements:**

All newborns had a dried blood spot card for TSH measurement 48 hours after birth as part of New Zealand’s Newborn Metabolic Screening Programme. Forty-one neonates also had a heel prick serum sample at one week to measure thyroid-stimulating hormone (TSH), free thyroxine (FT4), and free triiodothyronine (FT3). Maternal urine iodine concentration (UIC) and TSH in the six months after OSCM HSG were retrieved from the SELFI study for analyses.

**Primary outcome:**

Incidence of hypothyroidism in the neonatal period.

**Results:**

There was no evidence of primary hypothyroidism on newborn screening (TSH 2-10 mIU/L). All neonates tested at one week had normal serum TSH, FT4, and FT3 levels. However, increasing maternal peak UIC levels during pregnancy were associated with lower TSH levels (p= 0.006), although also associated with lower FT4 levels (p=0.032).

**Conclusions:**

While pre-conceptional OSCM HSG in women did not result in neonatal hypothyroidism, gestational iodine excess was associated with a paradoxical lowering of neonatal TSH levels despite lower FT4 levels. These changes likely reflect alterations in deiodinase activity in the fetal hypothalamic-pituitary axis from iodine excess.

**Trial registration:**

https://anzctr.org.au/Trial/Registration/TrialReview.aspx?ACTRN=12620000738921, identifier 12620000738921.

## Introduction

Oil-soluble contrast medium (OSCM) hysterosalpingography (HSG) improves pregnancy rates in women under 40 years of age (1, 2). However, Lipiodol Ultrafluide (Guerbet, Aulnay-Sous-Bois, France), the prototype OSCM has a high iodine concentration (480 mg/ml) and a long half-life (50 days) (3). Based on its pharmacokinetics, retention of OSCM in body compartments such as the peritoneum would be expected to result in severe and chronic iodine exposure (3). Indeed, this has been demonstrated in women undergoing OSCM HSG (4, 5). Recent data from our group have confirmed iodine excess is almost universal, with marked and prolonged iodine excess lasting more than six months (6). Most pregnancies following OSCM HSG occur within six months, and the majority within the first three cycles post-procedure (7, 8). If conception occurs while the iodine levels are high, there is an increased risk of iodine excess affecting the fetus, both directly and indirectly. Iodine exposure from skin disinfectants or contrast agents (9) is known to cause primary hypothyroidism in neonates, especially those born preterm. Similarly, iodine excess in mothers during late gestation from the diet (10), excessive multivitamin or iodine supplements (11, 12), or iodine-containing drugs such as amiodarone (13, 14) can cause primary hypothyroidism in newborns. The proposed mechanism is the Wolff–Chaikoff effect (9), whereby the iodine load inhibits thyroid peroxidase (TPO), blocking the synthesis and release of thyroid hormone (15). Unlike a normal adult thyroid gland, the preterm and fetal thyroid glands lack mechanisms to ‘escape’ from the Wolff–Chaikoff effect (16), which usually develops by 36–40 weeks of gestation (17). The result is the delayed recovery of thyroid function and potentially prolonged hypothyroidism in the fetus and newborn.

As OSCM HSG is becoming increasingly popular as a fertility-enhancing procedure, it is essential to establish its potential effects on neonatal thyroid function. Previous studies examining thyroid function in newborns conceived following an OSCM HSG had contradictory findings, with one showing an increased risk of primary hypothyroidism and the others showing no increased risk (5, 18–20). In a previous study by our research group, we analysed the Guthrie card thyroid-stimulating hormone (TSH) data of a separate cohort of 146 babies, who were retrospectively identified as having been conceived following an OSCM HSG between 2000 and 2019 in Auckland (New Zealand) (20). We observed no increase in the incidence of newborn hypothyroidism, including among newborns conceived in the immediate cycles following the HSG, when the iodine exposure would be maximal (20).

### Aims and objectives

This study aimed to prospectively determine the thyroid function status of the newborns conceived following OSCM HSG, and establish whether there were any associations between maternal iodine or thyroid hormone levels following the HSG and neonatal thyroid function. The objectives were to determine the incidence of:

1. Subclinical hypothyroidism in the newborn, defined as either: a) mild TSH elevation in the newborn screening (TSH 10–15 mIU/L) from dried blood spot cards (21, 22) on day 3; or b) TSH elevation above the age-appropriate lab reference range (0.4–16 mIU/L) with normal free thyroxine (FT4; 10–40 pmol/L) on the Day 7 serum sample.
2. Congenital hypothyroidism, defined as TSH >15 mIU/L from dried blood spot cards in the newborn screening and persistently elevated serum TSH levels or reduced FT4 in subsequent tests, requiring ongoing thyroxine replacement based on the stepwise complex protocol for diagnosis (23, 24)

## Materials and methods

**Figure 1:**
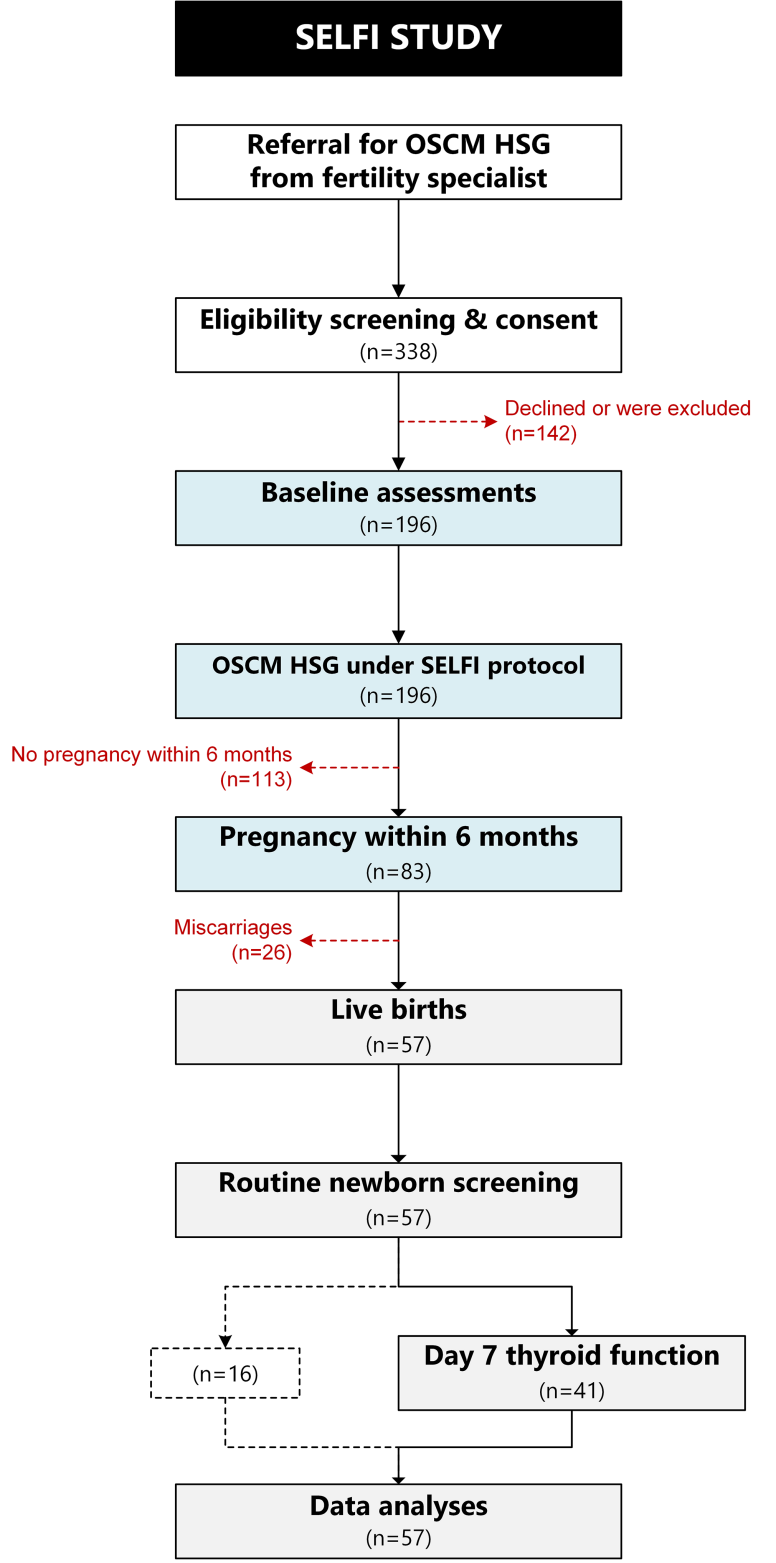
Flow diagram for the SELFI Study including the women who underwent oil-soluble contrast medium (OSCM) hysterosalpingography (HSG), those who became pregnant, and the subsequent number of newborns included in this study. Dashed black line indicates the newborns whose parents declined the Day 7 thyroid function test.

Participants were the offspring of the Safety and Efficacy of Lipiodol in Fertility Investigations (SELFI) study, conducted in Auckland. The study was approved by the Northern B Health and Disability Ethics Committee (Ministry of Health; 19/NTB/52) and registered with the Australian New Zealand Clinical Trials Registry (ANZCTR: 12620000738921). The aim of the SELFI study was to assess the magnitude and pattern of iodine excess and thyroid dysfunction following an OSCM HSG. The SELFI study cohort consisted of 196 consecutive consenting women who underwent OSCM HSG from July 2019 to April 2021, without overt hypothyroidism or hyperthyroidism, and who had not undergone recent hysterosalpingography. Participants had baseline assessments of urine iodine concentrations (UIC), TSH, FT4, and free triiodothyronine (FT3), and serial measurements of the same parameters for six months. More details on recruitment and assessments can be found in the published protocol (25) and the published results of maternal outcomes (6). Briefly, the SELFI study showed iodine excess (i.e., UIC >300 µg/L) in 98% of the women (6). Most participants had peak levels 1–12 weeks post-HSG, but elevated UICs persisted at 3-fold the maximum recommended levels even at 24 weeks post-HSG. Maternal TSH levels showed subtle elevation (mostly 4–10 mIU/L) with the peak TSH levels noted 1–12 weeks post HSG (6). A total of 83 participants (42%) had a biochemical pregnancy, of which 57 participants had a live birth. Details of the cohort and the flow of participants can be found in **Figure 1**.

All 57 babies had whole-blood TSH levels tested (from Guthrie cards) under New Zealand’s Newborn Metabolic Screening Programme 48 hours after birth. In addition, day 7 thyroid function tests were performed on newborns whose parents provided written informed consent for heel-prick sampling. The latter provided serum samples for thyroid function tests measuring concentrations of TSH, FT4, and FT3. No physical examinations were performed on these neonates, but data on sex, gestational age, birth anthropometry (weight, length, and head circumference), ethnicity, and mode of delivery were obtained from hospital records. Ethnicity of the newborn was reported by the parents, with a single ethnicity ascribed using the established hierarchical classification (26). Anthropometric measurements at birth were transformed into *z* scores adjusted for gestational age and sex using INTERGROWTH-21^st^ standards (27). The ponderal index was also calculated as per Röhrer’s formula [(100 x weight) ÷ length^3^], with weight in grams and length in cm. For this study, maternal data of interest included the time elapsed between HSG and pregnancy, and peak maternal UIC and TSH levels.

First-morning urine samples were used in mothers to measure UIC by inductively coupled plasma mass spectrometry (ICP-MS) using Agilent 7700 [coefficient of variation (CV) of 10.8%]. The assay for the newborn screening TSH was the GSP™ Neonatal hTSH (CV-8%). Serum TSH, FT4, and FT3 concentrations were measured with an electrochemiluminescence immunoassay using an ADVIA Centaur XP analyzer (Siemens Healthcare Diagnostics Inc., Tarrytown, NY, USA), with CVs ≤5% (28).

### Statistical analyses

Pearson’s correlation coefficients (*r*) were used to examine potential linear associations between maternal peak UIC or peak TSH levels and birth outcomes (i.e., gestational age, anthropometry *z* scores, and ponderal index), day 2 TSH levels, and day 7 TSH, FT4, and FT3 levels. The associations between each predictor and day 7 thyroid function parameters were also visualized in scatter plots, including the line of best fit from a simple linear regression and its coefficient (r^2^). The magnitude of statistically significant correlations was then quantified using general linear regression models, with results reported as the β coefficients and respective 95% confidence intervals (CI).

Additional general linear models were run for all offspring outcomes, including both maternal predictors (i.e., peak UIC and TSH levels) and their interaction term. If the latter was statistically significant, the interaction between the two predictors and a given outcome was illustrated with a contour plot.

Peak UIC levels were log-transformed to approximate a normal distribution. Statistical analyses were run using SAS v9.4 (SAS Institute, Cary, NC, USA). All statistical tests were two-tailed, with significance set at p<0.05.

## Results

All 57 newborns were screened by the national programme, but 16 parents declined the thyroid function tests on their babies at day 7 (**Figure 1**). However, the subgroup of 41 (72%) babies with a day 7 test was representative of the overall newborn cohort based on their demographic and birth characteristics (**Table 1**). Most neonates were conceived soon after the HSG, increasing the likelihood of being affected by maternal iodine exposure. Half of the recorded pregnancies (n=28) occurred within eight weeks of the HSG, and all but seven within four months (**Supplementary Figure 1**).

**Table 1:** Demographic and clinical characteristics of the offspring born in the SELFI study who were screened under the New Zealand newborn metabolic screening programme and those who also had a thyroid function test (TFT) at Day 7.

Characteristics	Levels	Newborn screening	Day 7 TFT
***n***		57	41
**Maternal age at baseline (years)**		33.9 ± 3.5	33.9 ± 3.5
**Sex**	**Female**	31 (54%)	20 (49%)
	**Male**	26 (46%)	21 (51%)
**Birth weight (kg)**		3.32 ± 0.48	3.34 ± 0.49
**Birth weight *z*-score**		0.41 ± 0.96	0.36 ± 0.98
**Gestational age (weeks)**		38.8 ± 1.4	39.0 ± 1.2
**Preterm birth ^1^**		3 (5%)	1 (2%)
**Head circumference (cm) ^2^**		34.7 ± 1.8	34.8 ± 1.8
**Head circumference *z*-score ^2^**		0.91 ± 1.26	0.94 ± 1.31
**Ponderal index**		2.52 ± 0.27	2.51 ± 0.28
**Ethnicity**	**European**	35 (61%)	27 (66%)
	**Indian**	11 (19%)	6 (15%)
	**Asian**	10 (18%)	8 (20%)
	**Māori**	1 (2%)	nil
**Type of delivery ^3^**	**LSCS**	27 (52%)	19 (49%)
	**NVD**	16 (31%)	13 (33%)
	**Ventouse**	7 (13%)	6 (15%)
	**Forceps**	2 (4%)	1 (3%)

Data are the mean ± standard deviation or n (%).

LSCS, lower segment caesarean section; NVD, normal vaginal delivery; TFT, thyroid function test.

^1^ Delivery at <37 weeks of gestation.

^2^ n=48 and n=37.

^3^ n=52 and n=39.

Thyroid function tests were performed on time at day 7 in 76% of subjects and within nine days in 90%. The remaining four tests were performed between 11 and 20 days. The delay from the proposed date was caused by the government-imposed lockdowns during the COVID-19 pandemic (29, 30). The results of the newborn screening tests on day 2 (whole-blood TSH) and Day 7 thyroid function tests (serum TSH, FT4, and FT3) are reported in **Table 2**. All 57 newborns had newborn screening (dried blood spot) TSH levels at day 2, and all babies tested had normal (age-appropriate) values for serum TSH, FT4, and FT3 on Day 7 thyroid function tests (**Table 2**). While the normative range for thyroid function changes over the first month of life, the appropriate ranges were used for the four delayed samples for Day 7 thyroid function tests. Of note, the Day 2 dried blood spot TSH levels are whole blood. Assuming a neonatal hematocrit of 0.5, serum TSH levels would be approximately double the dried blood spot levels.

**Table 2:** Results of thyroid function tests in the SELFI Study offspring.

Characteristics	*n*	Median [Q1, Q3]	Normal range
**Day 2 TSH (mIU/L) ^1^**	57	2.0 [1.0, 3.0]	0-15^4^
**Day 7 TSH (mIU/L) ^2^**	41	2.9 [2.1, 3.6]	0.4-16 ^5^
**FT4 (pmol/L) ^2,3^**	39	25.0 [21.0, 27.6]	10-40 ^5^
**FT3 (pmol/L) ^2,3^**	39	7.2 [6.4, 8.4]	3-10 ^5^

FT3, free triiodothyronine; FT4, free thyroxine (tetraiodothyronine); Q1, quartile 1 (25^th^ percentile); Q3, quartile 3 (75^th^ percentile); and TSH, thyroid-stimulating hormone.

^1^ Whole-blood TSH from Guthrie cards performed as part of the New Zealand National Screening Programme.

^2^ Serum values from thyroid function tests were performed within the SELFI Study.

^3^ FT4 and FT3 results were not available for two babies with inadequate samples to perform these tests.

^4^ Heather et al. Evaluation of the revised New Zealand national newborn screening protocol for congenital hypothyroidism. Clin Endocrinol 2017;86:431–7.

^5^ Reference range for TFT assays at Labtests, Auckland, where the tests were performed. Serum TSH, FT4, and FT3 were measured with an electrochemiluminescence immunoassay using a Siemens ADVIA Centaur XP analyzer.

**Figure 2:**
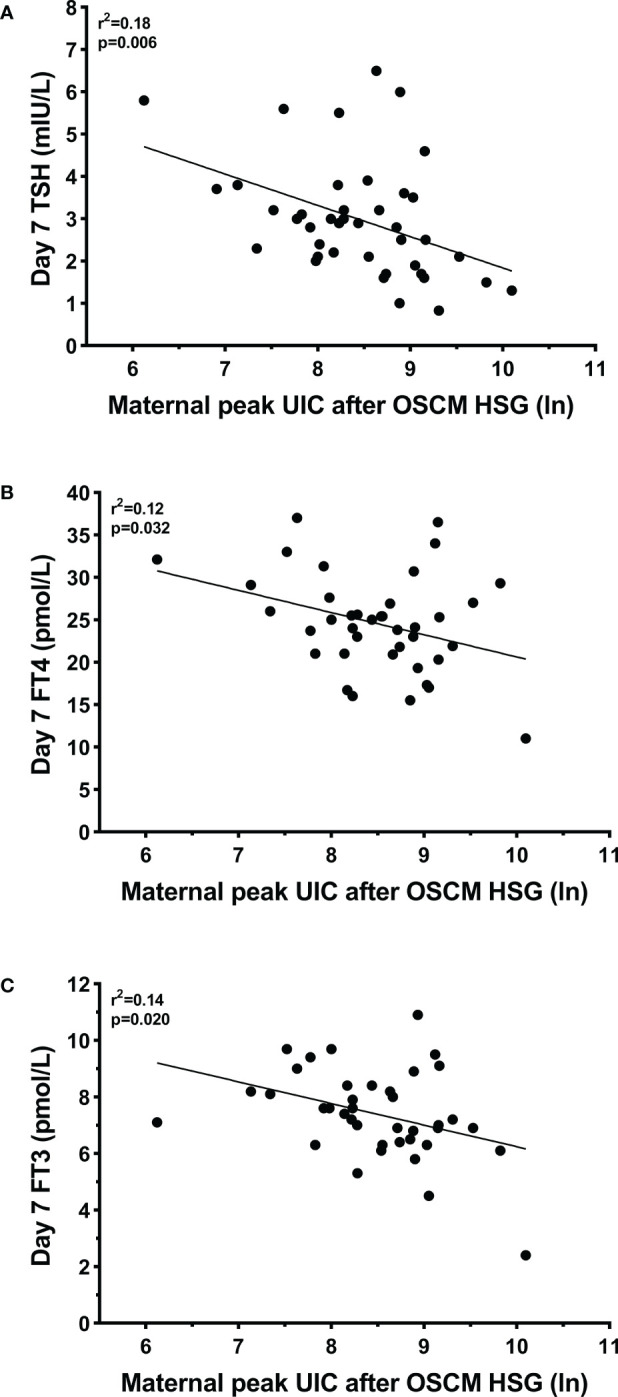
Linear associations between maternal peak urine iodine concentrations (UIC) during pregnancy and Day 7 thyroid function parameters (serum) in the offspring: **(A)** thyroid-stimulating hormone (TSH); **(B)** free thyroxine (tetraiodothyronine – FT4); and **(C)** free triiodothyronine (FT3). Peak UIC values were log-transformed to approximate a normal distribution. Diagonal grey lines are the fitted linear regressions, with their respective coefficients (r^2^) and *p*-values provided within each panel. HSG, hysterosalpingography; and OSCM, oil-soluble contrast medium.

There were no observed associations between maternal peak TSH or peak UIC and offspring parameters at birth or on Day 2 TSH (**Table 3**). However, on the Day 7 tests, increasing maternal peak UIC levels during pregnancy were associated with lower serum levels of all thyroid function parameters measured (**Table 3**, **Figure 2**). Specifically, a 50% increase in maternal peak UIC levels was associated with reductions of -1.1 pmol/L in FT4 (95% CI -2.0, -0.1 pmol/L; p=0.032) and -0.3 pmol/L in FT3 (95% CI -0.6, -0.1 pmol/L; p=0.020). While lower FT4 and FT3 levels would usually be associated with higher TSH values, this was not the case in this cohort, as paradoxically, the same 1.5 fold increase in maternal peak UIC was associated with a -0.3 mIU/L reduction in Day 7 TSH (95% CI -0.5, -0.1 mIU/L; p=0.006)].

**Table 3:** Linear associations between maternal urine iodine concentrations (UIC) and peak thyroid-stimulating hormone (TSH) levels during pregnancy and offspring outcomes.

		Maternal peak UIC			Maternal peak TSH		
Timing	Parameter	*n*	*r*	*p*	*n*	*r*	*p*
**Birth**	**Gestational age (weeks)**	57	-0.21	0.12	57	-0.20	0.13
	**Birth weight *z*-score**	57	0.26	0.05	57	0.02	0.91
	**Birth length *z*-score**	48	0.05	0.76	48	-0.02	0.88
	**Head circumference *z*-score**	48	0.02	0.91	48	0.05	0.74
	**Ponderal index**	48	0.13	0.37	48	0.03	0.86
**Postnatal day 2**	**TSH (mIU/L)**	57	0.13	0.33	57	0.18	0.19
**Postnatal day 7**	**TSH (mIU/L)**	41	**-0.42**	**0.006**	41	-0.28	0.08
	**FT4 (pmol/L)**	39	**-0.34**	**0.032**	39	-0.23	0.16
	**FT3 (pmol/L)**	39	**-0.37**	**0.020**	39	**-0.39**	**0.014**

Data are the Pearson’s correlation coefficients (r) and respective p-values, with statistically significant associations at p<0.05 shown in bold.

UIC values were log-transformed to approximate a normal distribution.

FT3, free triiodothyronine; FT4, free thyroxine (tetraiodothyronine); and TSH, thyroid-stimulating hormone.

Maternal peak TSH levels during pregnancy were also associated with lower Day 7 FT3 levels in the offspring (**Table 3**, **Supplementary Figure 2**). Specifically, an increase of 1.0 mIU/L in peak TSH was associated with a -0.3 pmol/L reduction in Day 7 FT3 concentrations (95% CI -0.6, -0.1 pmol/L; p=0.014).

When potential interactions between maternal peak TSH and peak UIC during pregnancy were examined, there was evidence of an interaction between those two parameters and their associations with Day 7 serum FT3 (p=0.018), but not TSH (p=0.87) or FT4 (p=0.62). For FT3, the lowest Day 7 levels were observed in the offspring of mothers with both high peak TSH and high peak UIC (**Supplementary Figure 3**).

## Discussion

This prospective offspring cohort study examined neonatal screening TSH data of 57 newborns whose mothers received pre-conceptional OSCM HSG, finding no cases of transient subclinical hypothyroidism or permanent congenital hypothyroidism. This is reassuring and is consistent with studies from China (5), the Netherlands (18), and New Zealand (20), which also assessed thyroid function status in the offspring using data from newborn screening programmes. We speculate that the declining iodine levels after the procedure would have resulted in lower maternal iodine levels in most women in late pregnancy, consequently reducing potential impacts on newborn thyroid function (6).

The only study that had previously shown an increased risk of primary hypothyroidism in newborns conceived following OSCM HSG was from Japan, where 2.4% of screened neonates (5/212) had an abnormal newborn screening result, and 0.94% (2/212) had primary hypothyroidism, noting that the background rate of congenital hypothyroidism in Japan is only 0.07% (31). The reasons for these conflicting results are unclear, but might be explained by continued iodine exposure from iodine-rich dietary sources in late pregnancy in Japan. The other common causes of gestational iodine excess such as maternal amiodarone therapy, dietary iodine excess, and use of topical iodine (10, 12, 14), often continue into late pregnancy and cause newborn primary hypothyroidism by a prolonged Wolff–Chaikoff effect. Although the latter was not observed in this cohort, a period of fetal hypothyroidism in early gestation from the Wolff–Chaikoff effect is theoretically possible with OSCM HSG, and could impact fetal development.

The association of maternal iodine levels with newborn’s Day 7 thyroid function was an unexpected finding, suggesting that iodine excess might have a direct, long-term effect on fetal thyroid function or fetal thyroid hormone regulation. The possibility of low T3 syndrome was considered less likely because there were no risk factors in our cohort, such as prematurity, asphyxia, sepsis, or other factors predisposing to a sick euthyroid status. Interestingly, similar findings in the offspring of rats receiving comparable doses of excess iodine during pregnancy have recently been shown with low TSH and low normal FT4 and FT3 (32). We speculate that the low TSH level reflects central alteration in thyrotropin-releasing hormone (TRH) and TSH regulation. Rodent studies have demonstrated altered hypothalamic and pituitary regulation, likely secondary to increased intracellular FT3 concentrations. Alterations in hypothalamic deiodinase 2 (an enzyme converting T4 to T3) and increased MCT8 receptor (a selective T3 transporter) expression, have been shown to reduce TRH secretion (33). Thus, the finding that women with higher UIC in early pregnancy had offspring with lower TSH and FT4 levels might be explained by this effect of iodine excess on the hypothalamo-pituitary axis. Whether these changes are permanent and/or have later life implications remains to be determined.

Overall, primary thyroid dysfunction in newborns is not a complication following maternal OSCM HSG. However, some newborns, especially those exposed to higher levels of iodine, appear to have subtle changes in the hypothalamic-pituitary-thyroid axis, and further studies are required to confirm and assess whether these changes are present at older ages. There is also a possibity for transient fetal hypothyroidism from iodine excess, which recover by birth and could not have be assessed in the current study. For the above reasons, neurocognitive assessments of these children would be required to ascertain whether there are any long-term developmental issues.

The main limitation of our study was the lack of a control population to compare the incidence of similar subtle changes in thyroid function. While our study population was not particularly large (57 newborns), it is difficult to prospectively study a larger number of offspring because pregnancy occurs only in a relatively small proportion of women who undergo HSG. Nonetheless, the present work is the only prospective investigation confirming normal neonatal thyroid status by serum thyroid function testing in the offspring conceived after OSCM HSG. Another key strength of this study was the availability of maternal parameters, allowing us to examine potential associations with offspring thyroid function and not performed in any previous studies. As there is widespread and growing popularity for the use of OSCM HSG as an infertility investigation and treatment modality, identifying the potential impacts of the associated iodine excess on the offspring is of clinical importance to inform specialists and infertile couples about the potential impacts of this intervention.

## Conclusions

This study confirms there is no increase in neonatal primary hypothyroidism in the offspring conceived following a standard OSCM HSG procedure. However, subtle central dysregulation of thyroid function may have occurred in some of these offspring. Future studies should be directed to assess the persistence of these changes in thyroid function and the potential neurodevelopment effects on these children.

## Data availability statement

The raw data supporting the conclusions of this article are not publicly available, but are available from the corresponding author on reasonable request and following the appropriate ethics approval.

## Ethics statement

The study was approved by the Northern B Health and Disability Ethics Committee, New Zealand (Ministry of Health; 19/NTB/52). Written informed consent to participate in this study was provided by the participants’ legal guardian/next of kin.

## Author contributions

PH, NJ, JP, RS, and SO’S conceptualized the study; DM conducted the study and drafted the initial manuscript; NH and DW assisted with newborn data collection, which were analysed by JD, DM, and PH. All authors contributed to the article and approved the submitted version.

## References

[1] Dreyer K van Rijswijk J Mijatovic V Goddijn M Verhoeve HR van Rooij IAJ. Oil-based or water-based contrast for hysterosalpingography in infertile women. New Engl J Med (2017) 376(21):2043–52. doi: 10.1056/NEJMoa1612337 28520519

[2] Johnson NP Farquhar CM Hadden WE Suckling J Yu Y Sadler L. The FLUSH trial–flushing with lipiodol for unexplained (and endometriosis-related) subfertility by hysterosalpingography: a randomized trial. Hum Reprod (2004) 19(9):2043–51. doi: 10.1093/humrep/deh418 15271870

[3] Miyamoto Y Tsujimoto T Iwai K Ishida K Uchimoto R Miyazawa T. Safety and pharmacokinetics of iotrolan in hysterosalpingography. retention and irritability compared with lipiodol. Invest Radiol (1995) 30(9):538–43. doi: 10.1097/00004424-199509000-00005 8537211

[4] Kaneshige T Arata N Harada S Ohashi T Sato S Umehara N. Changes in serum iodine concentration, urinary iodine excretion and thyroid function after hysterosalpingography using an oil-soluble iodinated contrast medium (lipiodol). J Clin Endocrinol Metab (2015) 100(3):E469–72. doi: 10.1210/jc.2014-2731 25546154

[5] Li R Chen W Liu Y Ma L Qiu L Han J. The impact of preconceptional hysterosalpingography with oil-based contrast on maternal and neonatal iodine status. Reprod Sci (2021) 28(10):2887–94. doi: 10.1007/s43032-021-00640-0 34080176

[6] Mathews DM Peart JM Sim RG Johnson NP O'Sullivan S Derraik JGB. The SELFI study: Iodine excess and thyroid dysfunction in women undergoing oil-soluble contrast hysterosalpingography. J Clin Endocrinol Metab (2022) 107(12):3252–60. doi: 10.1210/clinem/dgac546 PMC9693785 36124847

[7] Johnson NP. Review of lipiodol treatment for infertility - an innovative treatment for endometriosis-related infertility? Aust New Z J Obstetr Gynaecol (2014) 54(1):9–12. doi: 10.1111/ajo.12141 24138402

[8] Reindollar RH Regan MM Neumann PJ Levine BS Thornton KL Alper MM. A randomized clinical trial to evaluate optimal treatment for unexplained infertility: the fast track and standard treatment (FASTT) trial. Fertil Steril (2010) 94(3):888–99. doi: 10.1016/j.fertnstert.2009.04.022 19531445

[9] l'Allemand D Grüters A Beyer P Weber B. Iodine in contrast agents and skin disinfectants is the major cause for hypothyroidism in premature infants during intensive care. Hormone Res (1987) 28(1):42–9. doi: 10.1159/000180924 3447940

[10] Nishiyama S Mikeda T Okada T Nakamura K Kotani T Hishinuma A. Transient hypothyroidism or persistent hyperthyrotropinemia in neonates born to mothers with excessive iodine intake. Thyroid (2004) 14(12):1077–83. doi: 10.1089/thy.2004.14.1077 15650362

[11] Thomas Jde V Collett-Solberg PF. Perinatal goiter with increased iodine uptake and hypothyroidism due to excess maternal iodine ingestion. Hormone Res (2009) 72(6):344–7. 10.1159/000249162 19844123

[12] Connelly KJ Boston BA Pearce EN Sesser D Snyder D Braverman LE. Congenital hypothyroidism caused by excess prenatal maternal iodine ingestion. J Pediatr (2012) 161(4):760–2. doi: 10.1016/j.jpeds.2012.05.057 PMC4354797 22841183

[13] Aguilar Diosdado M Jáen Amate F Gavilán Villarejo I Merino López FJ. [Neonatal hypothyroidism after amiodarone therapy during pregnancy]. Anales Med Interna (1992) 9(7):358. 1633246

[14] Lomenick JP Jackson WA Backeljauw PF. Amiodarone-induced neonatal hypothyroidism: a unique form of transient early-onset hypothyroidism. J Perinatol (2004) 24(6):397–9. doi: 10.1038/sj.jp.7211104 15167882

[15] Leung AM Braverman LE. Consequences of excess iodine. Nat Rev Endocrinol (2014) 10(3):136–42. doi: 10.1038/nrendo.2013.251 PMC3976240 24342882

[16] Markou K Georgopoulos N Kyriazopoulou V Vagenakis AG. Iodine-induced hypothyroidism. Thyroid (2001) 11(5):501–10. doi: 10.1089/105072501300176462 11396709

[17] Chung HR. Iodine and thyroid function. Ann Pediatr Endocrinol Metab (2014) 19(1):8–12. doi: 10.6065/apem.2014.19.1.8 24926457 PMC4049553

[18] van Welie N Roest I Portela M van Rijswijk J Koks C Lambalk CB. Thyroid function in neonates conceived after hysterosalpingography with iodinated contrast. Hum Reprod (2020) 35(5):1159–67. doi: 10.1093/humrep/deaa049 PMC7259368 32427280

[19] Satoh M Aso K Katagiri Y. Thyroid dysfunction in neonates born to mothers who have undergone hysterosalpingography involving an oil-soluble iodinated contrast medium. Hormone Res Paediatr (2015) 84(6):370–5. doi: 10.1159/000439381 26402613

[20] Mathews DM Peart JM Johnson NP Sim RG Heather NL Webster D. Hysterosalpingography with oil-soluble contrast medium does not increase newborn hypothyroidism. Int J Endocrinol (2022) 2022:4532714. doi: 10.1155/2022/4532714 35242184 PMC8888087

[21] West R Hong J Derraik JG Webster D Heather NL Hofman PL. Newborn screening TSH values less than 15 mIU/L are not associated with long-term hypothyroidism or cognitive impairment. J Clin Endocrinol Metab (2020) 105(9):e3329–e38. doi: 10.1210/clinem/dgaa415 32598474

[22] Lain SJ Wiley V Jack M Martin AJ Wilcken B Nassar N. Association of elevated neonatal thyroid-stimulating hormone levels with school performance and stimulant prescription for attention deficit hyperactivity disorder in childhood. Eur J Pediatr (2021) 180(4):1073–80. doi: 10.1007/s00431-020-03828-9 33057816

[23] Heather NL Hofman PL de Hora M Carll J Derraik JG Webster D. Evaluation of the revised New Zealand national newborn screening protocol for congenital hypothyroidism. Clin Endocrinol (2017) 86(3):431–7. doi: 10.1111/cen.13250 27696498

[24] Albert BB Cutfield WS Webster D Carll J Derraik JG Jefferies C. Etiology of increasing incidence of congenital hypothyroidism in New Zealand from 1993-2010. J Clin Endocrinol Metab (2012) 97(9):3155–60. doi: 10.1210/jc.2012-1562 22723332

[25] Mathews DM Peart JM Sim RG Johnson NP O'Sullivan S Derraik JGB. The effect of acute and chronic iodine excess on thyroid profile and reproductive function of women using lipiodol during hysterosalpingography and the potential impact on thyroid function of their offspring: The SELFI study protocol. Med: Case Rep Study Protoc (2021) 2(8):e0148. doi: 10.1097/MD9.0000000000000148

[26] Ministry of Health. HISO 10001:2017 Ethnicity Data Protocols. Wellington: Ministry of Health, vol. 43. (2016).

[27] Villar J Cheikh Ismail L Victora CG Ohuma EO Bertino E Altman DG. International standards for newborn weight, length, and head circumference by gestational age and sex: the newborn cross-sectional study of the INTERGROWTH-21st project. Lancet (2014) 384(9946):857–68. doi: 10.1016/S0140-6736(14)60932-6 25209487

[28] Barth JH Spencer JD Goodall SR Luvai A. Reference intervals for thyroid hormones on advia centaur derived from three reference populations and a review of the literature. Ann Clin Biochem (2016) 53(Pt 3):385–9. doi: 10.1177/0004563216636897 27048695

[29] Baker MG Wilson N Anglemyer A. Successful elimination of Covid-19 transmission in New Zealand. New Engl J Med (2020) 383(8):e56. doi: 10.1056/NEJMc2025203 32767891 PMC7449141

[30] Sowman-Lund S. Auckland’s almost-four-month lockdown, in numbers. New Zealand: The Spinoff (2021). Available at: https://thespinoff.co.nz/society/03-12-2021/aucklands-almost-four-month-lockdown-in-numbers.

[31] Gu YH Kato T Harada S Inomata H Saito T Aoki K. Seasonality in the incidence of congenital hypothyroidism in Japan: gender-specific patterns and correlation with temperature. Thyroid (2007) 17(9):869–74. doi: 10.1089/thy.2006.0317 17956160

[32] Lebsir D Guemri J Kereselidze D Grison S Benderitter M Pech A. Repeated potassium iodide exposure during pregnancy impairs progeny's brain development. Neuroscience (2019) 406:606–16. doi: 10.1016/j.neuroscience.2019.02.016 30797025

[33] Sun Y Du X Shan Z Teng W Jiang Y. Effects of iodine excess on serum thyrotropin-releasing hormone levels and type 2 deiodinase in the hypothalamus of wistar rats. Br J Nutr (2022) 127(11):1631–8. doi: 10.1017/S0007114521002592 34250878

