# Supplementary material for: The impact of prolonged, maternal iodine exposure in early gestation on neonatal thyroid function
Source: Front Endocrinol (Lausanne). 2023 Jan 31;14:1080330. doi: 10.3389/fendo.2023.1080330 (PMC9927197; doi:10.3389/fendo.2023.1080330)
Supplement: Supplementary file 1 [file DataSheet_1.pdf]

Supplementary Files

The impact of prolonged, maternal iodine exposure in early gestation on neonatal thyroid function

Divya M Mathews[1,2*], Jane M Peart[3], Robert G Sim[3], Susannah O'Sullivan[4], José G B Derraik[1,5,6,7], Natasha L Heather[1,8], Dianne Webster[1,8], Neil P Johnson[9,10,11], Paul L Hofman[1,2]

[1] Liggins Institute, University of Auckland, Auckland, New Zealand

[2] Starship Children's Hospital, Te Whatu Ora – Health New Zealand, Te Toka Tumai Auckland, Auckland, New Zealand

[3] Auckland Radiology Group, Auckland, New Zealand

[4] Endocrinology, Greenlane Clinical Centre, Te Whatu Ora – Health New Zealand, Te Toka Tumai Auckland, Auckland, New Zealand

[5] Department of Paediatrics: Child & Youth Health, Faculty of Medicine and Health Sciences, University of Auckland, Auckland, New Zealand

[6] Department of Women's and Children's Health, Uppsala University, Uppsala, Sweden

[7] Environmental-Occupational Health Sciences and Non-Communicable Diseases Research Group, Research Institute for Health Sciences, Chiang Mai University, Chiang Mai, Thailand

[8] Newborn Metabolic Screening Programme, Lab Plus, Te Whatu Ora – Health New Zealand, Te Toka Tumai Auckland, Auckland, New Zealand

[9] Robinson Research Institute, University of Adelaide, Adelaide, SA, Australia

[10] Department of Obstetrics and Gynecology, Faculty of Medical and Health Sciences, University of Auckland, Auckland, New Zealand

[11] Repromed Auckland, Auckland, New Zealand

***Correspondence:** Dr Divya Mathews, Liggins Institute, University of Auckland, Private Bag 92019, Auckland 1142, New Zealand; phone: +61 432 258 345; email: d.mathews@auckland.ac.nz; ORCID: 0000-0002-5163-1588

Mathews DM, Peart JM, Sim RG, Johnson NP, O'Sullivan S, Derraik JGB, Heather N, Webster D, Hofman PL. The impact of prolonged, maternal iodine exposure in early gestation on neonatal thyroid function. *Front Endocrinol* 2023;14:1080330. doi: 10.3389/fendo.2023.1080330.

Supplementary Figure 1

Timing of pregnancies among women who underwent oil-soluble contrast medium (OSCM) hysterosalpingography (HSG) in the SELFI Study and had a live birth.

The number above each bar indicates the cumulative number of pregnancies at a given time-point.

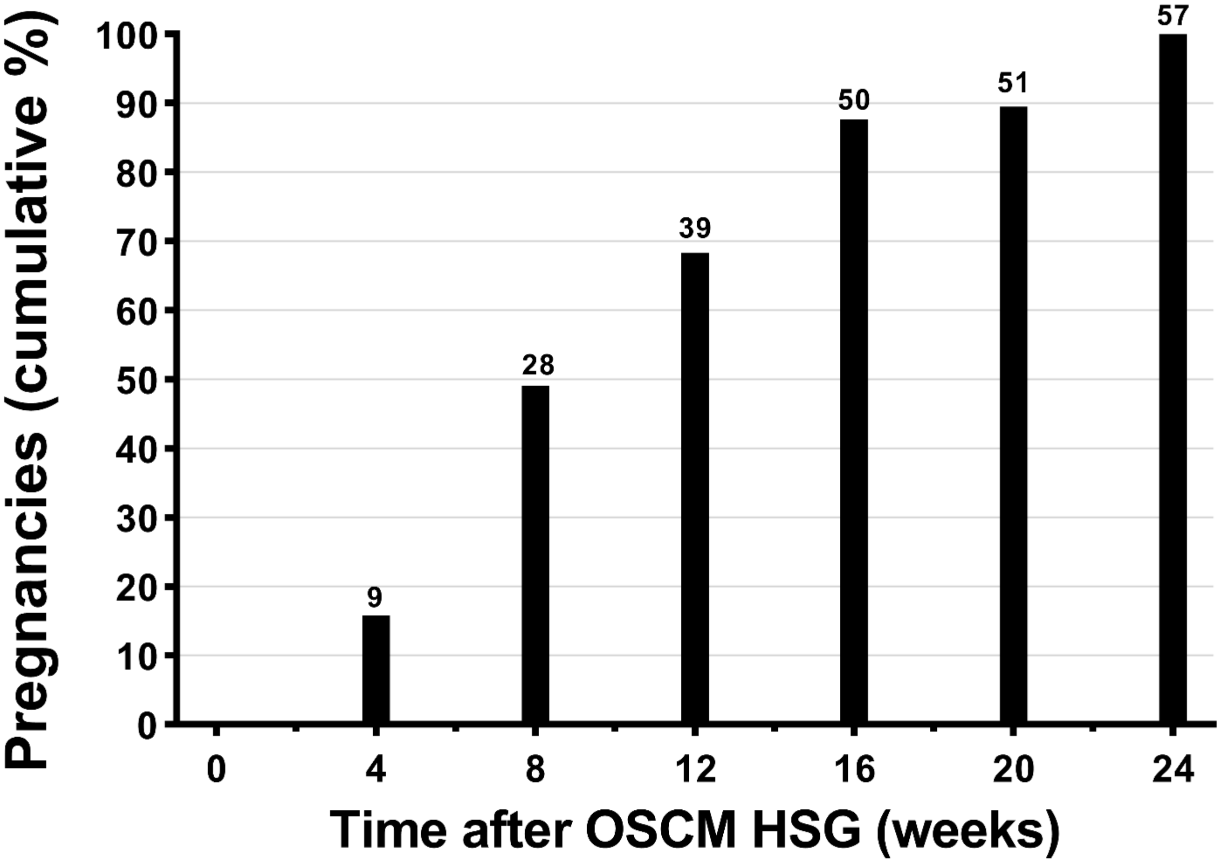

Mathews DM, Peart JM, Sim RG, Johnson NP, O'Sullivan S, Derraik JGB, Heather N, Webster D, Hofman PL. The impact of prolonged, maternal iodine exposure in early gestation on neonatal thyroid function. *Front Endocrinol* 2023;14:1080330. doi: 10.3389/fendo.2023.1080330.

Supplementary Figure 2

Linear associations between maternal peak thyroid-stimulating hormone (TSH) levels during pregnancy and Day 7 serum thyroid function parameters in the offspring: A) TSH; B) free thyroxine (tetraiodothyronine – FT4); and C) free triiodothyronine (FT3).

Diagonal grey lines are the fitted linear regressions, with their respective coefficients (r^2) and *p*-values provided within individual panels. HSG, hysterosalpingography; and OSCM, oil-soluble contrast medium.

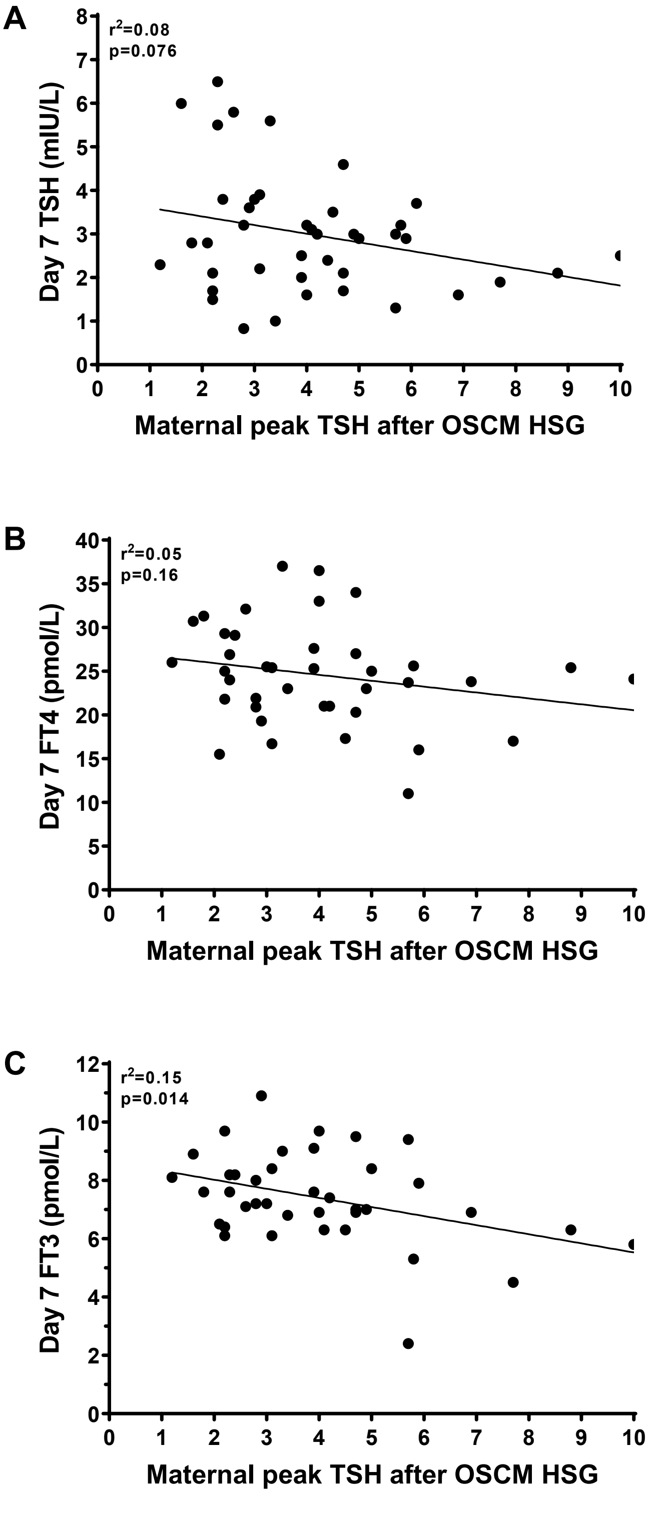

Mathews DM, Peart JM, Sim RG, Johnson NP, O'Sullivan S, Derraik JGB, Heather N, Webster D, Hofman PL. The impact of prolonged, maternal iodine exposure in early gestation on neonatal thyroid function. *Front Endocrinol* 2023;14:1080330. doi: 10.3389/fendo.2023.1080330.

Supplementary Figure 3

Contour plot showing the interaction between maternal peak thyroid-stimulating hormone (TSH) levels and peak urine iodine concentrations (UIC) during pregnancy and their association with Day 7 free triiodothyronine (FT3, expressed in pmol/L) in the offspring.

Peak UIC values were log-transformed to approximate a normal distribution. Each dot corresponds to a mother-child pair in the SELFI study.

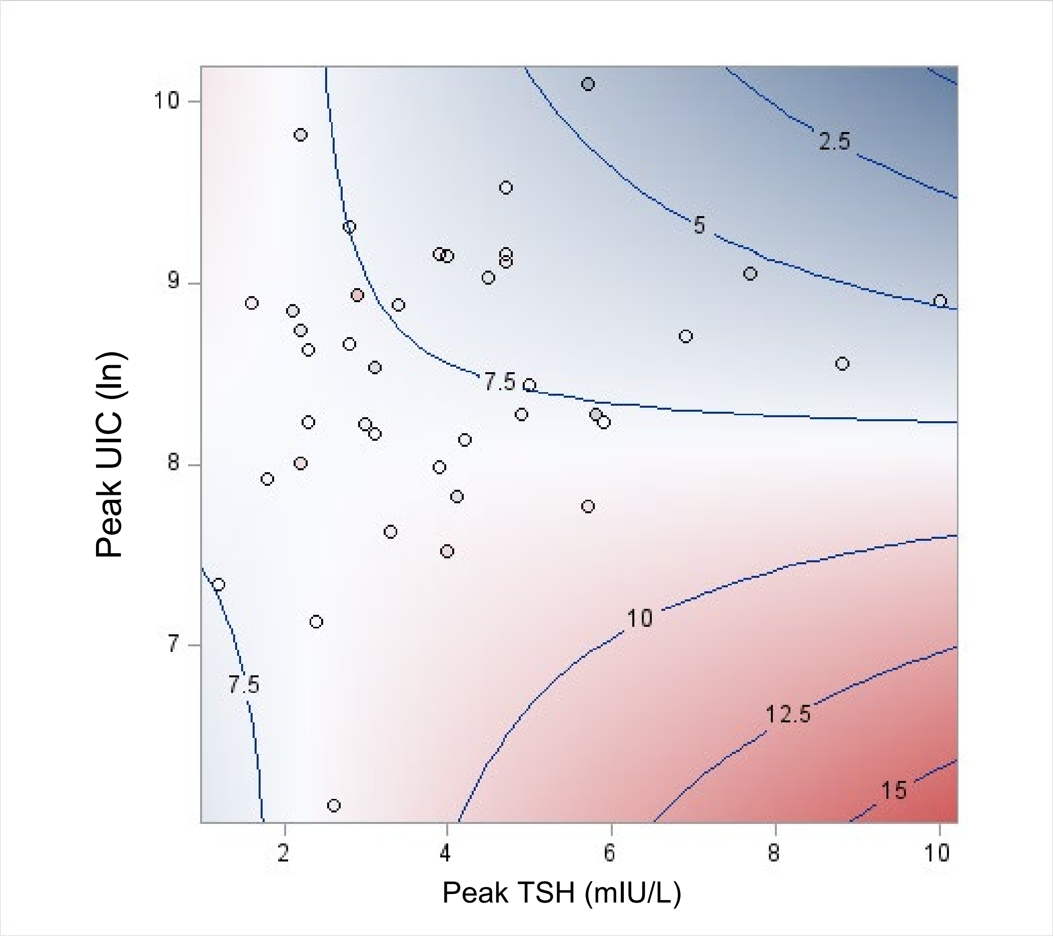

Mathews DM, Peart JM, Sim RG, Johnson NP, O'Sullivan S, Derraik JGB, Heather N, Webster D, Hofman PL. The impact of prolonged, maternal iodine exposure in early gestation on neonatal thyroid function. *Front Endocrinol* 2023;14:1080330. doi: 10.3389/fendo.2023.1080330.
